# Advancing Health Security and Disease Eradication Through Peace and Health: A Mali Case Study

**DOI:** 10.1089/hs.2023.0091

**Published:** 2024-04-16

**Authors:** Angelia M. Sanders, Madeline Warman, Frederic Deycard, John Goodman, April Klein, Karmen Unterwegner, Boukary Sangare, Sadi Moussa, Stacia George, Irene Pujol Chica, Cheick Oumar Coulibaly, Moussa Saye, Kimberly A. Jensen, Adam J. Weiss, Kashef Ijaz

**Affiliations:** Angelia M. Sanders, DrPH, MPH, MA, is Associate Directors, Trachoma Control Program; at The Carter Center, Atlanta, GA.; Madeline Warman, MA, is a Program Associate, in the Conflict Resolution Program; at The Carter Center, Atlanta, GA.; Frederic Deycard, PhD, is an Associate Director, in the Conflict Resolution Program; at The Carter Center, Atlanta, GA.; John Goodman, PhD, JD, is Director, Strasbourg Center, Syracuse University, Strasbourg, France.; April Klein, MPH, is a Graduate Assistant, in the Conflict Resolution Program; at The Carter Center, Atlanta, GA.; Karmen Unterwegner, MPH, is an Associate Director, Guinea Worm Eradication Program; at The Carter Center, Atlanta, GA.; Boukary Sangare, MA, is a Field Coordinator, Conflict Resolution Program; at The Carter Center, Bamako, Mali.; Sadi Moussa, MPH, is Senior Country Representative, Health and Peace Programs; at The Carter Center, Bamako, Mali.; Stacia George, MA, is Director, in the Conflict Resolution Program; at The Carter Center, Atlanta, GA.; Irene Pujol Chica, MA, is a Contractor, in the Conflict Resolution Program; at The Carter Center, Atlanta, GA.; Cheick Oumar Coulibaly, MD, MPH, is National Program Coordinator, Guinea Worm Eradication Program, Ministry of Health, Bamako, Mali.; Moussa Saye, MD, is a Technical Advisor, Guinea Worm Eradication Program; at The Carter Center, Bamako, Mali.; Kimberly A. Jensen, MPH, is Associate Directors, Trachoma Control Program; at The Carter Center, Atlanta, GA.; Adam J. Weiss, MPH, is Director, Guinea Worm Eradication Program; at The Carter Center, Atlanta, GA.; Kashef Ijaz, MD, MPH, is Vice President, Health Programs; at The Carter Center, Atlanta, GA.

**Keywords:** Mali, Guinea worm eradication, Conflict resolution, Health, Peace-health, One Health

## Abstract

Conflict and violence constitute threats to public health. As levels of conflict increase within and between countries, it is important to explore how conflict resolution initiatives can be adapted to meet the health needs of communities, and how addressing the health needs of communities can assist in conflict resolution and contribute to health security. In conflict-affected central Mali, a Peace through Health Initiative, piloted between 2018 and 2022, used conflict resolution trainings, facilitated community meetings, and human and animal health interventions to negotiate “periods of tranquility” to achieve public health goals. Project activities resulted in improved health, improved livelihoods, reduced violence, improved trust among stakeholders, and greater inclusion of community members in peace and health decisionmaking. The Peace-Health Initiative generated several lessons learned related to 3 phases of peace-health programming: preintervention, program development, and implementation. These lessons can be applied to support expanded Peace through Health Initiatives within Mali, may be adaptable to other conflict-afflicted contexts, and should be considered in relation to the implementation of global health security.

## Introduction

The concept of global health security centers on the belief that strong and resilient public health systems are needed to prevent, detect, and respond to infectious disease threats around the world.^1^ The Global Health Security Agenda is a global effort to strengthen the world's health institutions as related to laboratory and surveillance systems, workforce development, and emergency management and response.^2^ Successful implementation of this global agenda will require engaging hard-to-reach populations in regions or countries impacted by conflict and insecurity. At the end of 2022, approximately 108.4 million people worldwide were displaced as a result of conflict.^3^ Global health entities, such as the World Health Organization and the American Public Health Association, have recognized that conflict and violence constitute threats to public health and, thus, require a response from the public health community.^4-6^ There is evidence to suggest a cyclical cause-and-effect relationship between violence, poor population health, and stymied or inequitable economic development, which, in turn, perpetuates tensions and prolongs conflict.^7-10^ This relationship supports the characterization of violence as a social determinant of health.^11^ Countries experiencing armed conflict also experience relatively higher rates of morbidity and mortality.^12^ Additionally, armed conflict frequently limits health practitioners' access to affected populations in need of health services, as health workers and facilities can become targets of violence in these contexts.^13^

Better collaboration between the fields of conflict resolution and public health presents an opportunity to contribute to reducing the frequency of various types of violent events, improving the health of affected populations, and fostering positive, sustainable peace. Health, as a superordinate goal that transcends the political, ethnic, and social underpinnings of conflict, can serve as an entry point for peace, act as a bridge between stakeholders of the conflict, and foster cooperation toward a shared interest.^14,15^ The concept of superordinate goals is common in conflict resolution strategies.^16^ Health-focused superordinate goals carry great potential in conflict resolution; they not only elicit humane values but also represent an opportunity for mutual benefit, if provided in an equitable and impartial fashion with buy-in from stakeholders.^17^ Cooperation toward the wellbeing of civilians could build population support for the actors involved in a conflict and open additional channels of communication that support trust-building and peace.

Despite examples of successful peace through health initiatives, there has been limited documentation of strategy integration or collaboration between the fields of public health and conflict resolution in countries experiencing ongoing armed conflict. One documented example is the Sudan Guinea worm ceasefire in 1995, when the Sudanese government's Guinea Worm Eradication Program, supported by the nongovernmental organization (NGO) The Carter Center, used the primary goal of eradicating Guinea worm disease in Sudan as a basis for negotiating a ceasefire with relevant parties.^18,19^ The initial ceasefire was intended to last 2 months, but it essentially lasted 6 months and was the longest recorded humanitarian ceasefire at the time. During that time, thousands of cases of Guinea worm disease were identified, thousands of local community health workers were trained in disease surveillance and prevention measures, and polio and measles vaccination coverage increased.^20^

Drawing on the successful ceasefire negotiated around Guinea worm disease in Sudan, and The Carter Center's overall experience pursuing disease eradication in conflict and postconflict contexts, The Carter Center, in partnership with the Mali Ministry of Health, later implemented a Peace through Health Initiative in select locations of central Mali. In this case study, we describe the methods used, results achieved, and lessons learned from the first phase of implementation. These lessons may be applicable to other areas of Mali as well as other country contexts where insecurity negatively impacts health outcomes.

## Initiative Background

Mali has been impacted by violence since 2012, when a Tuareg-led rebellion and subsequent coup enabled violent jihadist groups to seize control in the country's central and northern regions. While the government has regained some territory, central Mali has seen exponential growth in violence since 2015.^21^ Military operations by the Malian army and international forces, armed attacks by militias and jihadi-linked groups, and regular use of improvised explosive devices are common occurrences. Massacres of civilians and gross violations of human rights are increasing in frequency.^22^ More than 311,000 people have been internally displaced, mostly to other parts of the central region, and the provision of essential services, including public health, have been reduced because of the violence.^23^

The climate of violence in rural central Mali has led to the flight and prolonged absence of government officials and services, including the Ministry of Health. Prior to the outbreak of violence in the region in 2015, the local population already held longstanding grievances about government corruption and lack of services.^21^ Indeed, the government's relative neglect of the area, combined with prevalent antigovernment sentiment and poverty, resulted in a population that was highly susceptible to violence and recruitment by radical armed groups.^24^ Today, the negative cycle of lack of services, resentment, violence, poverty, and withdrawal of government officials in central Mali is deeply entrenched and worsening.^25^

The Carter Center began direct support to Mali's Guinea Worm Eradication Program in 1993.^26^ Led by the Ministry of Health, the program team has reduced Guinea worm disease from 12,011 human cases in 1993 to zero human cases and 41 animal cases in 2022.^27,28^ Though previously widespread in the country, recent infections among domestic dogs and cats, as well as human cases of the disease, are believed to be linked to just 2 regions in central Mali: Mopti and Ségou. Villages with Guinea worm disease in Mopti are directly affected by violence, and many of those in Ségou are connected to violence occurring elsewhere through population movements. This makes thorough epidemiological investigations extremely difficult across all communities affected by Guinea worm disease. Furthermore, a cornerstone of Guinea worm eradication efforts is active community-based surveillance, including consistent daily visits that are rendered unsafe in communities affected by violence. For Mali to receive certification by the independent International Commission for the Certification of Dracunculiasis Eradication as being free of Guinea worm disease, its surveillance system must be able to quickly and consistently investigate rumors as a means of detecting every case and animal infection.^29^

When implementation of the Peace through Health Initiative began in 2018, its core objectives were to (1) improve access for government health workers, Carter Center staff, and other partners in an area where access had previously been limited or blocked entirely due to insecurity; (2) include vulnerable minority populations, such as young people and women, in the decisionmaking process and implementation of health interventions; (3) strengthen the local population's trust in government representatives; and (4) improve trust within local communities affected by intercommunal conflict.

## Initiative Implementation

**Figure 1.**
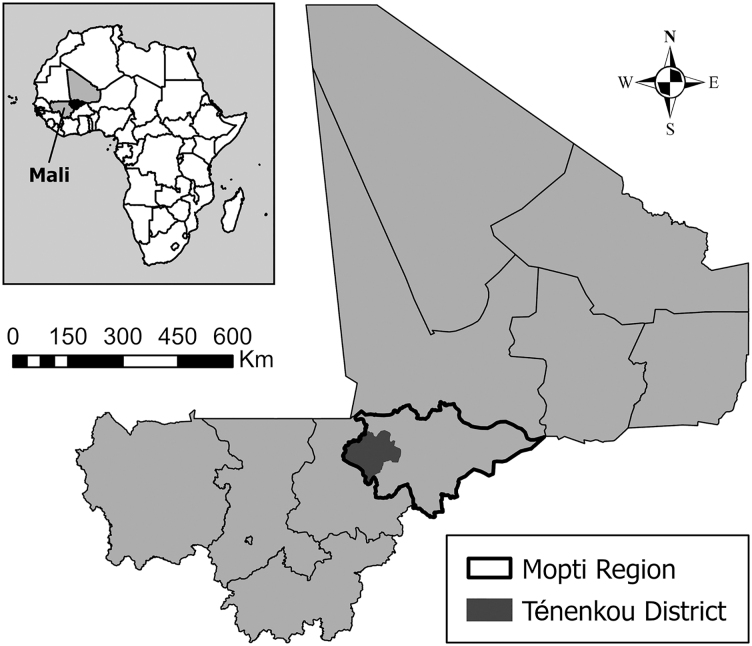
Ténenkou District, Mopti Region, Mali, 2022.

The Peace-Health Initiative focused on a limited target area in the Mopti region of central Mali (Figure 1). During the pilot phase, the specific target area was Ténenkou District, chosen because it was the suspected site of origin for some infections of Guinea worm disease in Mali and had been largely inaccessible to government actors. Guinea Worm Eradication Program activities, including oversight of village-level surveillance and the reporting and investigation of Guinea worm rumors, had been severely curtailed since 2012 due to insecurity and lack of trust between local communities and the Malian government. The Peace-Health Initiative's pilot phase, from 2018 to 2022, covered 5 health areas in the Ténenkou health district with a total population of about 40,000 people.

### Community Engagement

The Peace-Health Initiative did not involve classic diplomacy and conflict resolution methods used between governments; rather, it was based on grassroots, locally driven conflict mitigation and trust-building activities. This community-based, bottom-up approach focused on organizing dialogues on conflict resolution, inclusion, gender sensitivity, and shared public health concerns. The dialogues were between government health officials and community members living in areas beyond government control (ie, where government officials faced death or other security threats from jihadi-linked groups); armed actors did not participate. These dialogues and work sessions, which included women and other culturally or socially marginalized groups, resulted in the joint conception of health packages, while strengthening participants' conflict resolution skills.

### Health Packages

During the negotiated “periods of tranquility,” stakeholders (ie, government officials, health workers, and community members) collaborated on designing “health packages,” or sets of cocreated health services, provisions, and activities of interest to all. These health packages were then jointly implemented in coordination with the government and NGO partners. Each health package consisted of a set of activities identified by stakeholders as priorities and implemented by the Peace-Health Initiative and its partners through an approach sensitive to the conflict dynamics in the area. Within each health package, particular focus was placed on women's public health needs, such as maternity clinics, access to emergency transport during labor difficulties, and access to water. To engage stronger community buy-in, veterinary-related components to the package were also considered, in addition to other development-related needs, such as infrastructure.

### Implementation Process

The first step in the Peace-Health Initiative, starting in 2018, was an on-the-ground situational analysis to understand the conflict at the local level and develop a strategy tailored to the context. From October 2020 to May 2021, trainings on mediation, conflict resolution, and gender sensitivity were conducted for government workers and community representatives. These trainings formed the basis for further community dialogue related to the contents of the health package and ways to reduce violence to implement the health package. As part of piloting the Peace-Health Initiative, informal surveys and group discussions were conducted at various points to understand stakeholders' perceptions of the initiative, the conditions of peace in their community, and their level of trust in government, health workers, and other external actors. These surveys were conducted (1) during and after community dialogue sessions, (2) during and after conflict resolution and gender sensitivity trainings, (3) prior to the delivery of the health package, and (4) after the delivery of the health package.

## Initiative Achievements

From May 2021 to January 2022, the agreed-upon health packages were implemented in peaceful, nonviolent conditions jointly planned by rural community members and government officials. Several health and livelihood activities were achieved, such as an increase in Guinea worm disease surveillance, provision of eye care and maternal services, and veterinary-related interventions (Table 1).

**Table 1.** Peace-Health Initiative Health and Livelihood Achievements

Outcome	Activity
Health	• 123 GWD rumors investigated in 2021, compared to zero investigations in 2020 • 8 supervisory visits by the GWEP in 2021, compared to 4 in 2020 • 11 visits by the Ministry of Health regional office in 2021, compared to 7 in 2020 • 62 villages surrounding Ténenkou safely visited by GWEP representatives • 1 maternity ward repaired • 14 motor-mounted canoes provided for evacuations from local clinics to the regional capital of Mopti • 1,000 patients screened for cataracts, 350 cataract operations, and 1 cataract education campaign • Distribution of COVID-19 safety materials • Education campaign on the link between human health and veterinary diseases
Livelihood	• Approximately 60,000 animals vaccinated • 1,875 bags (50 kg each) of supplemental livestock feed delivered

Abbreviations: GWD, Guinea worm disease; GWEP, Guinea Worm Eradication Program.

In addition to the programmatic activities conducted, the Peace-Health Initiative advanced 3 key aspects of peacebuilding: violence reduction, increased trust between stakeholders, and greater community inclusion. Over the course of the Peace-Health Initiative, over 400 community members and health workers were surveyed or participated in group discussions with The Carter Center team and the Regional Directorate of Health of Mopti. Table 2 includes data on perceived outcomes from a group discussion held in October 2021, following the delivery of the health package in Ténenkou. The 35 participants included key project stakeholders such as village chiefs; representatives from the Local Federation of Community Health Associations (district level), Community Health Associations (health area level), women and youth groups, and traditional communicator networks; and health center directors.

**Table 2.** Stakeholder Perceptions of Peace-Health Initiative Outcomes (N=35)

Outcome	Reported Perceptions	Participant Response Ratio
Violence reduction	• An average of 1 violent incident per month reported by local health workers over the period of the Peace-Health Initiative's activities, compared to >10 per month before June 2021 (90% reduction) • Perceived decrease in violence and/or increase in personal safety reported	• 60% of group participants (n=21)
Increased trust	• Improved perception of government actors and health workers • Positive perception of the Peace-Health Initiative's team and effort • Increased trust in “other” ethnic groups	• 60% of group participants (n=21) • 80% of group participants (n=28) • 69% of group participants (n=24)
Greater inclusion	• Increased involvement in decisionmaking in the pilot period • Documented engagement of marginalized groups	• 83% of group participants (n=30) • 11% women (n=4) and 14% from populations such as youth and ethnic minorities (n=5)

## Discussion

Overall, the Peace-Health Initiative (1) strengthened the capacity of government officials (especially public health officials) and representatives of local communities (including youth, social minorities, and women) to prevent and mitigate conflicts by bringing them together for trainings in dialogue, gender sensitivity, conflict prevention, and mediation; (2) increased trust between government officials and local communities through facilitated dialogues and joint trainings; and (3) advanced peace by fostering meaningful cooperation between government officials and previously alienated local rural communities through the cocreation and execution of health packages. The health packages were a crucial incentive for dialogue, and as a superordinate goal, created a common objective to foster collaboration. Without the promise of a health package, there would be limited buy-in to participate in dialogue. Further, executing the agreed activities of the health packages was key to fostering trust through mutual accountability, since all stakeholders committed to specific collaborative actions to address security and public health challenges. Those actions resulted in desperately needed public health services and simultaneously helped increase trust between government officials and rural communities. This trust could then serve as a foundation to support further dialogue on other, non-health-related issues.

**Figure 2.**
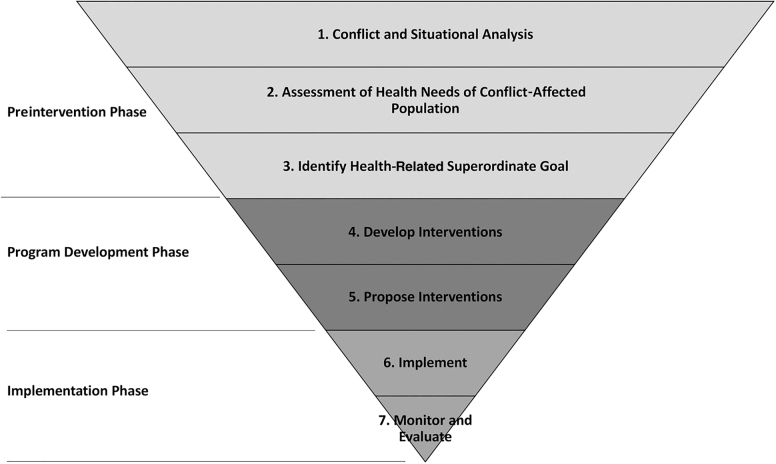
Peace-Health programming recommendations by phase of implementation.

Multiple lessons were learned throughout the Peace-Health Initiative's conceptualization, planning, and implementation processes. These lessons, discussed in the following sections, are presented as 7 recommendations that can serve as a guide for peacebuilding and public health practitioners to develop programs that are sensitive to a conflict context (Figure 2).

### Preintervention Phase

Before the intervention is implemented, it is important to (Recommendation 1) conduct a conflict and situational analysis. An analysis of the social, political, and ethnic underpinnings of the conflict, at the most local level possible, will help determine if a peace–health approach is appropriate. This is an area where public health actors would benefit from experts in conflict analysis or peacebuilding. The initial analysis can also help identify peace-specific indicators that could be monitored and evaluated during and after the intervention. Though this is the first step, the political and security context should be continually reassessed throughout the life of the project. Next, (Recommendation 2) assess the health needs of the population. Health needs assessments and surveillance, conducted by local or international actors, should be used to understand the major health disparities occurring within and between specific populations of concern and to leverage these issues for health-related peacebuilding. These health needs will also inform practitioners on what interventions are likely to be of mutual interest to community representatives, armed groups, and the government and thus establish a potential bridge for cooperation. As a final preintervention step, (Recommendation 3) identify health-related superordinate goals, which can help foster peace by serving as neutral starting points for bringing actors together to work toward joint objectives and create space for the prioritization of humane values.^17^

### Program Development Phase

As part of the program development phase, it is important to (Recommendation 4) develop interventions with both peace and health indicators. Peace–health scholars have claimed that health initiatives can influence the dynamics of a conflict, but there is a dearth of data on the peace outcomes of health interventions.^30^ This limits the ability to accurately state that peace–health initiatives have had any impact on long-term peace outcomes, aside from the obvious case of temporary ceasefires. Developing and monitoring indicators for peace outcomes could contribute to the evidence base for peace–health initiatives and lead to more robust evidence-based peace–health programs. Next, (Recommendation 5) propose interventions that emphasize both peace and health outcomes. The intervention program design should include expert input on implementation from both peacebuilding and public health practitioners. Collaboration at all stages and the identification of clear goals are crucial to ensuring that the intended peace and health impacts are realized.

### Implementation Phase

The key to the implementation phase is (Recommendation 6) to implement what has been discussed. The implementation should, whenever possible, include local health stakeholders—assuming they are perceived as impartial—in designing and delivering the health intervention(s). For example, if government entities are involved in implementation, the implementation process can help build trust in government institutions and increase sustainability. Concurrently, (Recommendation 7) commit to the monitoring and evaluation process as a fundamental component to peace–health interventions. While tracking health outputs and impacts may be as simple as quantifying the individuals reached and changes in incidence and prevalence rates of disease morbidity or mortality, measuring peace is less straightforward. Development of peace indicators should be informed by the situational analysis as well as the programmatic outcomes that may indicate reductions in existing tension and violence, resulting in improved tolerance and cooperation. Proxy indicators may be appropriate, and qualitative data and perceptions of change can be as informative as other measurements. The program should also include ongoing monitoring and reporting of violence, with contingency plans to suspend program activities for safety and security reasons.

### Limitations

The limitations to the initial implementation of the Peace-Health Initiative preclude any certainty that the work of the Initiative was the sole reason for improvement in community perceptions, particularly as there may have been other contributing factors happening in the region at the time. Although the Peace-Health Initiative tracked levels of violence and armed group presence remotely through conflict mapping prior to implementation, those same security concerns prevented any preimplementation surveys in local communities for the purpose of establishing a baseline. Health agents and community representatives did provide baseline trends, but their inexact nature made it a challenge to measure indicators such as changes in perceptions of government agents or freedom of movement with any certainty. To better evaluate the impact of the Peace-Health Initiative's approach, future programs should make every effort to include robust baseline surveys and participatory data collection with stakeholders, using sample sizes that could document statistical significance. Programs can use a network of local sources to collect data about distinct types of violence in zones of interest, which would allow for more tailored programming and a more comprehensive monitoring and evaluation framework.

Though not a limitation on the outcomes of the Peace-Health Initiative, fundraising for the negotiated health packages was ultimately a challenge. The Carter Center had funding available for peacebuilding and Guinea worm disease-related activities, but most of the other health interventions requested by the communities involved were not included in The Carter Center's portfolio for Mali. This meant that new funding sources and NGO partners and institutions had to be identified to provide the resources and expertise to deliver these components of the agreed health packages. It is recommended that future programs have some initial funding in place to quickly implement aspects of the negotiated health packages and ensure there are no delays in implementation. Delays or failures to implement the agreed health packages could undermine trust among stakeholders.

## Conclusion

In an era of epidemics and pandemics such as Ebola and COVID-19, increased emphasis has been placed on global health security both in the context of general health programming and specifically within the International Health Regulations^31^ and efforts to strengthen government preparedness and capacity.^32,33^ However, the growing number of conflicts worldwide, along with their increasingly protracted and complex nature, necessitate the development of equally complex solutions. The Global Health Security Agenda cannot be fully achieved without addressing public health needs for hard-to-reach populations. This case study describes interventions, lessons learned, and recommendations from the Peace-Health Initiative in Mali, that may be applicable to other populations that are hard to reach due to insecurity. By delivering concrete peace and health dividends through cross-sectoral collaborations, the Peace-Health Initiative fostered new relationships between stakeholders, a space and network for dialogue, a pattern and model of practice, and reductions in violence on which to build further conflict mitigation and peacebuilding activities. With careful adaptation to prevailing local contexts, the approach used in Ténenkou could be expanded to other districts in Mali and to other countries facing similar challenges.
